# Research Progress in the Effect of Traditional Chinese Medicine for Invigoration on Neurotransmitter Related Diseases

**DOI:** 10.1155/2018/4642018

**Published:** 2018-05-14

**Authors:** Xiting Zhang, Lin Li, Ting Chen, Zuoyan Sun, Weiwei Tang, Shuang Wang, Tianqi Wang, Yi Wang, Han Zhang

**Affiliations:** ^1^Institute of Traditional Chinese Medicine, Tianjin University of Traditional Chinese Medicine, Tianjin 300193, China; ^2^Key Laboratory of Pharmacology of Traditional Chinese Medical Formulae, Tianjin University of Traditional Chinese Medicine, Ministry of Education, Tianjin 300193, China

## Abstract

Tonic traditional Chinese medicine is widely used in clinical practice and is categorized into four main drugs, namely, Qi-supplementing, Blood-enriching, Yin-nourishing, and Yang-tonifying. Neurotransmitters play a coordinating role in the nervous system, visceral function, and stress response. The excitation or suppression of the central nervous system is closely related to various diseases, such as insomnia, depression, Alzheimer's disease, Parkinson's disease, and perimenopausal syndrome. An increasing amount of evidence shows that Chinese tonic herb and its active ingredients can delay the occurrence and development of these diseases by modulating related neurotransmitters and their receptors, including norepinephrine (NE), serotonin (5-HT), dopamine (DA), acetylcholine (ACh), and *γ*-aminobutyric acid (GABA). In the present report, studies on the treatment of these neurotransmitter related diseases in relation to the application of tonic Chinese medicine are reviewed.

## 1. Introduction

Tonic traditional Chinese herbal medicine can help in promoting the healthy well-being of organisms by replenishing their life essence. According to modern pharmacology research, tonic medicine can enhance immune capacity, strengthen learning and memory function, and regulate the endocrine function of patients with impaired disease. Interestingly, there are other beneficial effects, such as antiageing [1], antioxidant [2], and antistress [3] effects. According to its effect, tonic medicine is divided into Qi-tonifying, Blood-tonifying, Yin-tonifying, and Yang-tonifying prescriptions. Qi-tonifying prescriptions can tonify visceral-Qi to rectify the pathological deviation of the visceral-Qi deficiency [4]. For example, this may include invigorating the spleen and lungs to act on spleen and lung channels. Qi-tonifying prescription is used principally to cure representative medicines, including* Radix Ginseng*,* Acanthopanax senticosus, Rhodiola rosea, Astragalus, *and* Licorice*. Blood-tonifying prescriptions [5] target blood and heart deficiencies with such medicines as* Radix Paeonia Alba, Angelica sinensis*,* Polygonum multiflorum thumb, *and* Radix Rehmanniae.* Yin-tonifying prescription [6] is commonly applied to nourish Yin-fluid and correct the pathological deviation of Yin deficiency with such medicines as* Lily*,* Glossy Privet Fruit*, and* Eclipta alba*. In addition, Yang-tonifying prescriptions [7] can supplement Yang to treat all kinds of Yang deficiency diseases. These medicines can have sweet, pungent, salty, and warm properties and mainly affect the kidney channel. Medicines include* Psoralea corylifolia*,* Morinda officinalis*,* Cistanche tubulosa, Folium Epimedii, *and* Fructus Alpinia oxyphylla (see Table 1)*.

Recent findings indicate that tonic traditional Chinese herbal medicine can modulate neurotransmitters [8], improve insomnia, exert antidepressant effects, delay the development of neurodegenerative diseases, and improve the climacteric symptoms of women. In this paper, related research progress is reviewed* (see Table 2)*.

## 2. Methods

We searched PubMed using the following keywords: Related diseases (Depression/Alzheimer'sdisease/Parkinson's disease/Perimenopausal syndrome/Sleep disorders) AND Neurotransmitters (5-HT, DA, NE, ACh, GABA) AND Tonic traditional Chinese medicine (Representative drugs of Qi-supplementing, Blood-enriching, Yin-nourishing, and Yang-tonifying). We limit our search to studies only published in English language from PubMed. We also searched CNKI for Chinese articles with the same keywords. Time range we searched had no limitation (to present). In overall, we found 155 articles in the two databases, among which 70 were published in English and 85 were published in Chinese. 18 articles were excluded for repetition in the contents. According to the description of the contents such as Qi, blood, Yin, Yang, and the effective components of the tonic traditional Chinese medicine, we finally found 33 Chinese literatures and 44 English literatures.

Based on Chinese Materia Medica and Clinical common medicines, we selected the following representative drugs of tonic traditional Chinese medicine. (1) Qi-supplementing:* Radix Ginseng, Acanthopanax senticosus, Rhodiola rosea, Astragalus, *and* Licorice*; (2) Blood-enriching:* Radix Paeonia Alba, Angelica sinensis, Polygonum multiflorum thumb, *and* Radix Rehmanniae*; (3) Yin-nourishing:* Lily, Glossy privet fruit, *and* Eclipta alba*; (4) Yang-tonifying:* Psoralea corylifolia, Morinda officinalis, Cistanche tubulosa, Folium Epimedii, *and* Fructus Alpinia oxyphylla*.

## 3. The Relationship between Qi, Blood, Yin, Yang, and Neurotransmitters

Yin and Yang are two substances in the human body closely related to each other and their functional characters [9]. Yang refers to warmth, excitement, promotion, and so on, while Yin refers to nourishing, moistening, inhibiting, and so on. Yin and Yang stand against each other, can transform into each other, and rely on each other by operating in equilibrium. Working in equilibrium means that Yin and Yang have a balance of growth and decline and maintain dynamic balance in the changes of growth and decline [10]. If either side shall decrease or be insufficient, the other side would consequently be reduced or weakened [11].

Qi is an energetic subtle essence that forms the human body and sustains life activities [12]. Blood is a nourishing nutrient that runs in veins and circulates throughout the body. It is one of the basic substances that comprise the human body and sustains life activities. Qi and blood can transform into each other; that is, Qi can generate blood, and vice versa. Blood insufficiency will induce Qi deficiency and can cause mental diminishment, forgetfulness, insomnia, irritability, ability disorders, and other symptoms [13].

From the properties of Yin and Yang, we can determine that Qi possesses the properties of Yang, active and warm, whereas blood possesses the properties of Yin, static and moistening. They depend on and nourish each other to support and promote life activities [14].

The connection among Qi, blood, Yin, and Yang is achieved through the meridians [15]. Meridians are channels through which Qi and blood connect organs, body surfaces, and body parts. It works as a controlling system of human bodily functions by transporting Qi and blood to the entire body and nourishing tissues and organs. Meanwhile, meridians have similar physiological function to the nervous system. Meridian, neuroendocrine neurotransmitters, and receptors are closely linked. It is confirmed that sympathetic nerves, mast cells, calcium, adrenergic neurotransmitters, and other substances are related to meridian regulation. According to Chang et al. [16], the basis of meridian activity is a conditioned reflex with the involvement of the cerebral cortex and several researchers have discovered that there are a phenomena of neurotransmitter enrichments in the meridian sensing zone.

For example, in the study of meridian acupuncture points, Omura [17] discovered that most points have highly enriched neurotransmitters and hormones, including acetylcholine, norepinephrine, adrenocorticotropic hormone, 5-HT, and *γ*-GABA. During the study of signal conduction in acupuncture, Liu's research group discovered that acupuncture allows meridians to release norepinephrine, epinephrine, and other catecholamines along the skin, and that their content was significantly higher than nonacupunctured meridians [18, 19].

The weakness of Qi, blood, Yin, and Yang may affect neurotransmitters through meridians.

## 4. The Relationship between Tonic Traditional Chinese Medicine and Neurotransmitter Related Diseases

### 4.1. Depression

Depression is defined as a mental disorder characterized by psychomotor retardation, such as slow thinking, accompanied by decreased interest and initiative. Recent studies have demonstrated that a deficiency in the following neurotransmitters is correlated with the onset of depression: 5-HT, DA, NE, Ach, and GABA [20]. Importantly, most antidepressants exert antidepressant effects by increasing levels of neurotransmitters located at synapses.

Depression belongs to the category of “melancholia” in Chinese Medicine. Melancholia is a Chinese medicine syndrome caused by emotional discomfort and Qi-stagnation [21]. Chinese medicine has extensive experience in the prevention and treatment of depression. For example, such medicines invigorate the liver, spleen, kidney, and heart, but also the quiet spirit too. However, the combination of these medicines with antidepressant activity is a widespread concern.

The chronic mild unpredictable stress (CMUS) model is among the most common methods for studying depression [22]. It is primarily used for the screening of antidepressants and the pathophysiology of depression.

The forced swimming tests (FST) and tail suspension tests (TST) are well-established models for studying depression in animals [23, 24]. In the frontal cortex and hippocampus, FST was observed to indicate a significant decrease in 5-HT levels with a slight change in 5-HIAA levels, resulting in an elevation in the 5-HIAA/5-HT ratio [25].

### 4.2. Alzheimer's Disease (AD)

Alzheimer's disease (AD) is an insidious and progressive neurodegenerative disease that affects the nervous system. It is characterized by dementia and includes memory impairment, aphasia, and executive dysfunction. NE, DA, 5-HT, and cholinergic transmitter ACh are important neurotransmitters in the brain. Among them, choline acetyltransferase (ChAT) is a key enzyme in the synthesis of Ach [26], and AchE is a key enzyme in the degradation of ACh. Acetylcholinesterase inhibitor (AChEI) therapy is a frequently used method in the treatment of AD [27]. Both ChAT and AchE activities are strongly correlated with ACh content in the brain. They maintain the dynamic balance of ACh and exert important roles in maintaining normal cognitive function. A reduction in these neurotransmitters can lead to senile dementia. More specifically, studies show that NE modulates the cortex through axons and has broad implications for mood, cognitive function, and movement [28]. DA has been found to reduce oxidative stress, increase blood flow to the brain, and coordinate with NE to regulate the body's excitability. In addition, 5-HT modulates central cholinergic nerve activity by regulating the release of Ach in brain tissue, which is an important neurotransmitter for mediating intelligence. The decrease of Ach content is suggested to be closely related to the severity of clinical symptoms of AD [29].

In recent times, modern medicine has yet to produce an effective prevention and cure for AD. By contrast, traditional Chinese medicine has unique advantages because it is characterized by multiple targets and pathways. It holds the opinion that AD belongs to the category of dementia. Although the basic pathogenesis is symptoms caused by asthenia, kidney vacuity and marrow depletion are the basis of the disease [30]. In this regard, tonic traditional Chinese medicine may help to improve AD symptoms. Many reports from cell and animal experiments found that *β*-amyloid (A*β*) is the key protein in AD patients. The A*β* fragment peptide 1–42 (A*β*1–42) induced neuronal damage and apoptosis via oxidative stress* in vitro* [31, 32]. A*β*25–35 exhibits similar early neurotropic and late neurotoxic activities as A*β* [33]. Therefore, A*β*1–42 and A*β*25–35 are commonly used as a model method.

### 4.3. Parkinson's Disease (PD)

Parkinson's disease (PD) is another multicentral neurodegenerative disease predominated by movement retardation. It is a complicated and poorly understood pathogenesis, with clinical symptoms characterized by tremor, slowness of movement, and postural instability caused by various factors. The most significant change in the disease is the decrease in DA levels in the brain. Tyrosine hydroxylase (TH) is the rate-limiting enzyme responsible for catalyzing the conversion of the amino acid L-tyrosine to L-3,4-dihydroxyphenylalanine (L-DOPA) [34]. L-DOPA is a precursor for neurotransmitter DA. DA is divided into metabolites by a set of enzymes—monoamine oxidase (MAO) and catechol-O-methyl transferase (COMT). The final product of DA metabolism is DOPAC and HVA, both of which can be used as an indicator for the change of DA content in the brain [35].

Traditional Chinese medicine defines PD as belonging to a category of diseases, such as paralysis agitans, the basic pathogenesis of which involves the liver and kidney. Therefore, the use of Chinese herbal medicine may be helpful in improving and delaying the symptoms of the disease.

### 4.4. Sleep Disorders

Insomnia is a sleep disorder syndrome whereby sleep quality cannot meet individual physiological needs, resulting in fatigue, lack of attention, and a lag in mental response. Long-term insomnia is harmful to human physical and mental health and can cause multiple organ dysfunction and immune function decline related to hypertension, diabetes, and other mental disorders. The dysfunction of neurotransmitters such as 5-HT and DA are closely linked with insomnia. According to the theory of traditional Chinese medicine, palpitation, forgetfulness, insomnia, and tiredness are mostly deficiency syndromes. It is advisable to tonify the nerves and tranquilize the mind by tonifying traditional Chinese herbal medicine.

GABA is the major inhibitory neurotransmitter in CNS and is essential for mediating the overall balance between neuronal excitation and inhibition [36]. The molecular target of medicinal plants with a sedative hypnotic activity involves the benzodiazepine site of the GABAA (GABAA-BZD) receptor. GABAergic neurotransmission plays a key role in sleep regulation, and the BZD binding site on the GABAA receptor is a target for most sedative-hypnotics [37]. BZD agents, such as diazepam, can stimulate the ability of GABA to induce hyperpolarization of membranes by allowing a Cl^−^ influx.

### 4.5. Perimenopausal Syndrome

Perimenopausal syndrome is a series of syndromes characterized by autonomic dysfunction caused by the decline of ovarian function and the decrease of estrogen levels in women during and around menopause. Ovary dysfunction, imbalance of the hypothalamus pituitary gonadal axis, disorder of sex hormone secretion, and dysfunction of the autonomic nervous system in perimenopausal women may affect NE, DA, and 5-HT levels in the hypothalamus [38]. This, in turn, may lead to hot flashes, sweating, irritability, insomnia, and other symptoms. NE and DA are two neurotransmitters with extensive function in the brain and can regulate functional activities of several neurons and correct hyperactivity of central and peripheral sympathetic nerves. As an important neurotransmitter in the brain, decreased 5-HT levels can reduce impulses released by the body temperature regulating center, thereby helping to control the frequency and amplitude of hot flashes representative of menopausal syndrome (MPS). MPS may be improved to a certain extent via the adjustment and intervention of the above-mentioned neuroendocrine networks. The surgical removal of bilateral ovaries from female rats is the main method used for a simulation model of MPS. For example, studies have found that levels of DA, NE, 5-HT, and 5-HIAA in the hypothalamus were significantly increased in ovariectomized rats compared with those rats in the control group [39–41].

From the theory of traditional Chinese medicine, perimenopausal syndrome is characterized by deficiencies of the kidney, although dysfunctions of the liver, spleen, and heart are also common [42]. More specifically, the theory of syndrome differentiation is an important aspect of traditional Chinese medicine. According to this theory, not only is the condition of the kidney important, but also the condition of the other four major organs in combination with the condition of the five internal organs is important because this shows the complexity of pathological change. Medicines for tonifying deficiency have been shown to be satisfactory in the treatment of perimenopausal syndrome.

## 5. The Effects of Traditional Chinese Medicine on Neurotransmitters

### 5.1. Qi-Tonifying Drugs

The common representative medicines of Qi-tonifying drugs are* Rhodiola rosea*,* Acanthopanax senticosus*,* Radix Ginseng*,* Licorice*,* Panax quinquefolius*,* Astragalus membranaceus*, and so on.

The following herbs and active ingredients play antidepressant roles by affecting neurotransmitters.

*Rhodiola rosea* is of the genus* Rhodiola and* is a plant of the Crassulaceae family of succulent leaf plants. After 1.5, 3, and 6 g/kg treatment of* Rhodiola rosea*, 5-HT levels in male Sprague-Dawley (SD) rats were observed to be significantly higher than those of the CMUS group, whereas 5-HT levels in the medium and high dosage group were normal. In the low dosage group, however, 5-HT levels were observed to be significantly higher than those of the control group [43].

*Acanthopanax senticosus (*ASE) is of the genus* Eleutherococcus *and is a plant of the Araliaceae family of ivy plants.* A. senticosus *has been widely used in the treatment of mental disorders for many thousands of years. In terms of the FST, ASE at dosages of 500, 1000, and 2000 mg/kg were administered to male Kunming mice at a weight of 18–20 g over seven days, whereas ASE was observed to significantly reduce immobility time compared with the control group. Furthermore, ASE was observed to increase 5-HT levels and also significantly increased DA and NE levels in a dose-dependent manner [44].

*Licorice* is of the genus* Glycyrrhiza *and is a plant of the Leguminosae family of flowering plants. It has been reported that an aqueous extract of* G. glabra L.* showed significant antidepressant-like activity in mouse immobility tests [45]. A previous study found that a flavone compound, liquiritin, isolated from* G. uralensis *demonstrated an antidepressant effect on chronic stress depressed rats [46]. In the present study, liquiritin and another flavonoid from* G. uralensis*, isoliquiritin, were investigated to determine their antidepressant activities. Liquiritin and isoliquiritin are the active ingredients of* Licorice*. Liquiritin and isoliquiritin at dosages of 10, 20, and 40 mg/kg were observed to significantly decrease the immobility time in mice during FST and TST. At medium concentrations, liquiritin and isoliquiritin were both found to increase 5-HT and NE levels in the hippocampus, hypothalamus, and cortex in mice in both the FST and TST. However, liquiritin and isoliquiritin also significantly decreased the ratio of 5-HIAA/5-HT in all measured brain regions in both the FST and TST [47].

*Ginseng* is of the genus* Panax* and is a plant of the Araliaceae family of ivy plants.* Ginseng *has been used for mood adjustment in traditional Chinese medicine for thousands of years. The previous study has shown that, total ginsenosides, the major pharmacologically functional ingredients of ginseng, possess antidepressant activity [48]. In the present study, they hypothesized that an intestinal metabolite of ginseng, 20(S)-protopanaxadiol (code name S111), as a postmetabolism compound (PMC) of ingested ginsenosides, may be responsible for the antidepressant activity of ginseng. Male Swiss mice weighing 18–22 g were administered S111 at dosages of 3.75, 7.5, and 15 mg/kg over a treatment period of 10 days. The immobility time of the mice was observed to decrease in both the FST and TST. Furthermore, male SD mice at a weight of 200–250 g that underwent an olfactory bulbectomy (OB) were observed to have lower levels of NE and 5-HT than those mice who underwent a FST and TST. After treatment with S111 at 13.33 mg/kg and 6.67 mg/kg daily for 14 days, the levels of NE and 5-HT were significantly higher than the OB model group [49].

The following herbs and active ingredients may delay the development of PD by affecting the neurotransmitter.

*Acanthopanax senticosus* is of the genus* Eleutherococcus* and is a plant of the Araliaceae family of ivy plants. The extract of* Acanthopanax senticosus *was observed to have a neuroprotective effect against MPTP-induced PD model mice. MPTP can selectively damage neurons in the nigrostriatal dopaminergic pathway and cause PD in humans, nonhuman primates, and mice; thus, mice have become widely accepted as a model for PD [50, 51]. After 20 days of treatment, the DA level of striatum in the low dose group (45.5 mg/kg) was observed to significantly increase [52].

*Panax quinquefolius *is the genus of* Panax* and is a plant of the family Araliaceae of ivy plants. Pseudoginsenoside-F11 is a unique component and main monomer in* Panax quinquefolius* [53]. It has been shown to have many beneficial effects in treating disorders of 6-OHDA-lesioned rats. After treatment with PF11 at dosages of 3, 6, and 12 mg/kg, the level of striatal extracellular DA was observed to significantly increase in a dose-dependent manner compared with 6-OHDA-lesioned PD model rats. At a high dosage, extracellular DA was observed to recover to normal levels. Furthermore, a noticeable restoration of TH was observed in the herb-treated group in a dose-dependent manner [54].

*Astragalus* membranaceus is of the genus* Astragalus* and is a plant of the Leguminosae family of flowering plants.* A. membranaceus *is a medicinal plant traditionally used in Chinese medicine for several conditions, including inflammatory and neural diseases.* Astragalus *polysaccharides can reduce the adverse effects of levodopa and are used in the treatment of PD [55].* Astragalus* at dosages of 1, 2, or 4 mg/ml is capable of alleviating 6-OHDA-mediated dopaminergic neurodegeneration in BZ555 nematodes. AchE is an indirect indicator of cholinergic system function and provides a helpful strategy for alleviating the behavioral deficit in PD progression. After being exposed to 6-OHDA, AchE activity was observed to decrease in BZ555 nematodes. This decreased AchE activity was observed to be significantly elevated when treated with 2 mg/ml of astragalin, indicating that* Astragalus* can regulate AchE activity in BZ555 nematodes [56].

The following herbs and active ingredients may improve sleep disorders by affecting neurotransmitters.

*Licorice* is of the genus* Glycyrrhiza *and is a plant of the Leguminosae family of flowering plants.* Glycyrrhiza glabra* (GGE) is the root of* Glycyrrhiza *and is a frequently used natural medicine described as “the grandfather of herbs”. GGE was observed to decrease sleep latency and increase sleep duration in a dose-dependent manner (50, 100, 250, and 500 mg/kg). Its hypnotic effect was observed to show a statistically significant difference at concentrations of 250 and 500 mg/kg compared with the pentobarbital-induced model group. The hypnotic effect observed in this study suggests that GGE has the potential to exert positive allosteric modulation of GABAA-BZD receptors. The specific GABAA-BZD receptor antagonist flumazenil (FLU) was observed to significantly inhibit the hypnotic effects of GGE at a dosage of 500 mg/kg. This indicates that GGE induces sleep via the GABAergic system and can act as a positive allosteric modulator on GABAA-BZD receptors to activate glabrol compounds (50 mg/kg) [57].

*Eleutherococcus senticosus *is of the genus* Eleutherococcus *and is a plant of the Araliaceae family of ivy plants. Eleutheroside E (EE) is a principal component of* Eleutherococcus senticosus.* The world health organization (WHO) prescribes the anthocyanin E as one of the indicators of Acanthopanax quality control [58]. In addition, In China Pharmacopoeia 2010, the content of anthocyanin E in the Acanthopanax extract was determined [59]. Treatment with EE at dosages of 10 and 50 mg/kg was observed to improve behavioral tests of sleep deprived model mice, such as passive avoidance task locomotor activity and Y-maze testing. Furthermore, after 72-hour sleep deprivation and EE treatment at a dosage of 50 mg/kg, a significant increase in 5-HT concentration was observed. However, the EE (50 mg/kg) treatment group significantly decreased DA concentrations after sleep deprivation when compared to the control group [60].

### 5.2. Blood-Tonifying Drugs

The most common representative medicines of blood-tonifying drugs are* peony, Fallopia multiflora,* and so on.

The following active ingredients play an antidepressant role by affecting neurotransmitters.

*Peony* is of the genus* Paeonia lactiflora* and is a plant of the Ranunculaceae family of flowering plants. The previous studies found that the total glycoside fraction of peony exerted significant antidepressant-like effects in multiple animal models of depression [61, 62]. Paeoniflorin, the main active component in total glycosides of peony, has been extensively studied as an antioxidant, a cognitive enhancer or learning impairment-attenuating agent, and a neuroprotective agent related to depression. It has been observed to increase the sucrose consumption of CMUS-model rats. Compared with CUS rats, Paeoniflorin-H (60 mg/kg) can elevate levels of NA, DA, 5-HT, and 5-HIAA in such a way as to decrease the 5-HIAA/5-HT ratio in the central nervous system of CUS-model rats [63].

The following active ingredients may delay the development of PD by affecting the neurotransmitters.

As mentioned, Peoniflorin (PF) is a major bioactive ingredient in the roots of* Radix Paeonia Alba*. PF has a low toxicity and has been demonstrated to have neuroprotective effects. After treatment with PF at dosages of 7.5, 15, and 30 mg/kg, the level of DA and HVA in striatum were significantly higher than that of the MPTP-treated group, whereas the ratio of HVA/DA was lower than that of the model group. Dopaminergic transporter (DAT) plays a significant role in mediating the return of striatal dopamine. PF at dosages of 7.5, 15, and 30 mg/kg was observed to attenuate the decrease of DAT and TH in the striatum and the substantia nigra [64].

*Fallopia multiflora* is of the genus* Fallopia* and is a plant of the* Polygonaceae* family of knotweed plants. 2,3,5,4′-tetrahydroxystilbene-2-O-*β*-D-glucoside (TSG) is an active component extracted from this traditional Chinese herb. 2,3,5,4′-Tetrahydroxystilbene-2-O-*β*-D-glucoside (TSG) was isolated from* Fallopia multiflora*, a plant which is traditionally used as an antiageing drug. TSG at dosages of 20 and 40 mg/kg was observed to significantly increase the level of DA, DOPAC, and HVA in the striatum. TSG at dosages of 20 and 40 mg/kg was also observed to attenuate the loss of TH-positive cells induced by MPTP in a dose-dependent manner. A higher dosage was more effective than a lower dosage [65].

### 5.3. Yin-Tonifying Drugs

The most common representative medicines of Yin-tonifying drugs are* Eclipta, Glossy privet fruit, Lilium brownii, Rhizoma anemarrhenae, *and so on.

The following active ingredients may delay the development of AD by affecting the neurotransmitters.

*Eclipta compositae* is of the genus* Eclipta *and is a plant of the family Compositae of sunflower plants. A model of Alzheimer's disease was established with subcutaneous injections of D-galactose and microinjection A*β*25–35 on the bilateral hippocampus of male and female SD rats. Eclipta alba was observed to improve the learning and memory function of rats in the AD model. Compared with the AD model group, however, the incubation period was observed to be prolonged in the step-through test, with error times significantly decreased in the high dosage group (24 g/kg). Furthermore,* Eclipta alba* extract at dose of 24 g/kg was observed to significantly enhance the contents of NE, DA, and 5-HT in the hippocampus of AD model rats. In addition, a high dosage was also observed to increase ChAT activity and significantly decline AchE [66].

The following Yin-nourishing prescription improves symptoms of perimenopausal syndrome by affecting neurotransmitters.

ErzhiBaihe concentrated cream is used for the treatment of early stage of menopause, and consists of tonic herbs, including* Glossy privet fruit, Eclipta, Lilium brownii, *and* Rhizoma anemarrhenae*. Various concentrations of ErzhiBaihe cream (1.92, 0.96, 0.48 g/kg) were administered to the model rats and the level of NE, DA, and 5-HT in their hypothalamus was observed to decrease. The difference between the high dose group and the model group was also observed to be statistically significant. These results demonstrate that the Erzhi Recipe could rectify hypothalamic neurotransmitter disorders and efficiently regulate the endocrine disturbance of model rats [39].

### 5.4. Yang-Tonifying Drugs

The most common representative medicines of Yang-tonifying drugs are* Psoralea corylifolia*,* Morinda officinal*,* Cistanche tubulosa*,* Fructus Alpinia oxyphylla*,* Fructus Alpinia oxyphylla*, and so on.

The following active ingredients play an antidepressant role by affecting neurotransmitters.

*Psoralea corylifolia* is of the genus* Psoralea *and is a plant of the Leguminosae family of flowering plants. The previous studies demonstrated that the total furocoumarins extracts of* P. corylifolia* had the potent antidepressant properties by employing the forced swimming test (FST) [67]. Psoralen is a main furocoumarin isolated from the seeds of* Psoralea corylifolia*. Male mice of the ICR strain at a weight of 23–25 g were administered psoralen at dosages of 10, 20, and 40 mg/kg over a 14-day treatment period. At the end of treatment, immobility time was observed to significantly increase in the FST, with 20 and 40 mg/kg dosages significantly increasing swimming time. While psoralen treatment was not observed to affect the 5-HIAA/5-HT ratio, psoralen at a dosage of 20 mg/kg significantly increased 5-HT levels in the hippocampus and frontal cortex of the mice [68].

The following herbs and active ingredients may delay the development of AD by affecting the neurotransmitter.

*Morinda officinal *is of the genus* Morinda* and is a plant of the Rubiaceae family of flowering plants.* Morinda officinalis* mainly contains compounds and inorganic elements such as sugar, quinone, amino acids, lipids, and organic acids [69]. Among them, the content of oligosaccharide is up to 50%, the composition is complex, and the pharmacological action is obvious. Bajijiasu is a oligosaccharide monomer isolated from* Morinda officinalis*, and it has the activity of invigorating the kidney and brain, which can obviously improve the behavior of vascular dementia rats [70]. In the Alzheimer's disease (AD) model, male SD rats were injected with A*β*25–35 into the bilateral CA1 region of the hippocampus. Bajijiasu at dosages of 8, 24, and 48 mg/kg was observed to ameliorate A*β*-induced learning and memory dysfunction compared with the AD model group. The escape latency of each BJ-treated group was significantly shorter in the Morris water maze. After Bajijiasu was administered at different concentrations, the levels of Ach, 5-HT, and DA were found to significantly increase while AchE decreased [71].

*Cistanche tubulosa *(CT) is of the genus* Cistanche* and is a plant of the family Orobanchaceae of parasitic plants. All doses of CT (100, 200 mg/kg) extract significantly increased the time spent in the platform-quadrant of the Morris water maze and increased the step-through latency of the inhibitory avoidance task compared to A*β*1–42-infused male SD rats. A*β* 1–42 was observed to decrease the expression of DA and Ach in the hippocampus, which could be improved by the aqueous extracts of CT at a dosage of 200 mg/kg. Furthermore, CT extract (200 mg/kg) was observed to reduce ACHE and MAO-A cortical activity in the model group after 15 days treatment [72].

The following herbs and active ingredients may delay the development of PD by affecting the neurotransmitters.

*Fructus Alpinia oxyphylla *(AOE) is of the genus* Alpinia* and is a ripe fruit of the Zingiberaceae family of flowering plants. AOE was observed to increase locomotor activity in 6-OHDA-treated* Zebrafish*. Furthermore, AOE at dosages of 6 and 12 ug/ml was observed to significantly attenuate the loss of the DA Neuron of 6-OHDA induced in* Zebrafish*. Furthermore, the DA neuron almost completely recovered by giving high doses of AOE [73].

*Psoralea corylifolia *is of the genus* Psoralea *and is a plant of the Leguminosae family of flowering plants. The monoterpenoids are dominant components in the “volatile oils” of plant species and △^3^,2-hydroxy bakuchiol (BU) is an important monoterpene phenol compound in* Psoralea corylifolia* [74]. BU at dosages of 5.44, 16.32, or 48.96 mg/kg was observed to significantly inhibit the uptake of DA in striatal synaptosomes and NE in the hippocampal synaptosomes of male SD rats. BU at dosages of 4, 20, or 100 mg/kg was also observed to significantly inhibit the decrease of TH-positive neuronal populations in the substantia nigra (SN) of MPTP model male C57BL/6J mice [75].

The following active ingredients may improve sleep disorders by affecting neurotransmitters.

*Fructus Alpinia oxyphylla* is of the genus* Alpinia* and is a ripe fruit of the Zingiberaceae family of flowering plants. Glutamic acid (Glu) is a main excitatory neurotransmitter in the central nervous system (CNS). An increase in the release of Glu can induce excitatory neurotoxicity. The ratio of GABA/Glu plays a critical role in the balance of excitation and inhibition in neurons. Compared with the control group, the levels of Glu amino acid neurotransmitters were observed to increase, whereas GABA was significantly decreased in the cortex and hypothalamus during rapid eye movement (REM) sleep deprivation. However, the group of volatile oils extracted from* Alpinia oxyphylla Fructus* (VOA) (9, 27 g/kg) reversed the increase in Glu and decreased GABA compared to the model group [76].

### 5.5. Tonify Deficiency Prescriptions

The tonify deficiency prescriptions contain Qi-tonifying, Blood-tonifying, Yin-tonifying, and Yang-tonifying drugs which are compatible with each other.

The following prescription may delay the development of PD by affecting the neurotransmitters.

Jia-Jian-Di-Huang-Yin-Zi (JJDHYZ) decoction is a classical prescription of traditional Chinese medicine and contains* Radix Paeonia Alba, Angelica sinensis, Radix Rehmanniae, Morinda officinalis, *and* Cistanche*. JJDHYZ been used in the treatment of long-term neurological disorders. JJDHYZ at a high concentration of 34 g/kg was observed to improve the variation tendency in behavioral pole tests. Furthermore, the injection of MPTP was also observed to demonstrate neurotoxic effects and induce a reduction of dopamine (DA), homovanillic (HVA), and dihydroxyphenylacetic (DOPAC) acids compared with the control group. JJDHYZ at a concentration of 34 g/kg significantly rescued the depletion of DA, DOPAC, and HVA in comparison with the MPTP group [77].

The following prescriptions improve symptoms of perimenopausal syndrome by affecting neurotransmitters.

A herbal recipe derived from Danggui Buxue Decoction consists of* Radix astragali*,* Radix Angelica sinensis*, and* Folium Epimedii (*RRF), with a ratio of 5 : 1 : 5. The three are Yang-tonifying, Blood-enriching, and Qi-supplementing drugs, respectively. Medium concentrations (282 mg/kg/d) of RRF were observed to significantly inhibit increased hypothalamic NE in rats after they were ovariectomized, whereas high concentrations (564 mg/kg/d) of RRF were observed to significantly reduce the increased hypothalamic DA level in model rats. Finally, high and low concentrations (564 and 141 mg/kg/d) of RRF significantly reduced the increased hypothalamic 5-HT level in the model rats. However, levels of 5-HIAA and the 5-HT metabolite remained unaffected by different concentrations of RRF [40].

Bushen Liaogeng Extract (BLE) is effective in the clinical treatment of perimenopausal syndrome and is prepared from several deficiency-tonifying Chinese herbs, including* Radix Rehmanniae Praeparata, Cistanche deserticola, Cynomorium songaricum, *and* Codonopsis pilosula*. Compared with the model group, hypothalamic DA, NE, 5-HT, and 5-HIAA levels in mice from the low, medium, and high dose BLE groups (4.5, 9, and 18 g/kg) were significantly decreased. Therefore, this suggests that BLE can regulate the level of monoamine neurotransmitters in the hypothalamus of ovariectomized mice by restoring its normal status and improving disordered autonomic nervous function during menopause [41].

## 6. Summary and Future Prospects

The neurotransmitter related diseases such as depression, Alzheimer's disease, Parkinson's disease, sleep disorders, and perimenopausal syndrome are complicated disorders and their pathogenesis remains unclear. These diseases are closely correlated with the dysregulation of central neurotransmitters. At present, there exists no clinically effective medicines available for the treatment of these diseases given their obvious side effects. The theory of traditional Chinese medicine believes that the pathogenesis of such diseases is complicated, involving the Yin-Yang and the Qi-blood of five internal organs. Previous studies have demonstrated that tonic Chinese medicine can improve the symptoms of disease and prolong the development of diseases by supplementing Qi, enriching blood, nourishing Yin, and strengthening Yang. An increasing amount of studies have documented that tonic Chinese medicine plays a therapeutic role and can regulate the secretion of central nervous system neurotransmitters, such as 5-HT, DA, and NE by multicomponent, multichannel, and multitarget therapy. In the future, the exact target, location, and mechanism of the effective ingredient and function of tonic Chinese medicine should be investigated.

## Figures and Tables

**Table 1:** Modern Research about the function of common tonic traditional Chinese medicine.

Tonic Herbs	Represents	Properties	Channel tropism	Function	Modern research
Qi-invugorating	*Ginseng*	Sweet Slightly bitter Mild	Lung Spleen Heart	Excessive nourishing primordial qi. Tranquilization method.	Anti-inflammation Antiallergic Antifatigue
	*Acanthopanaxsenticosus*	Sweet Slightly bitter	Spleen Lung Heart Kidney	Nourishing qi to invigorate spleen. Invigorating kidney and tranquilization method.	Antifatigue Antistress Antiradiation Antihypoxia
	*Rhodiola rosea*	Sweet Cold	Spleen Lung	Invigorates spleen and nourishing qi. Promoting blood circulation for removing blood stasis.	Antifatigue Antihypoxia Improves brain activity
	*Astragalus*	Sweet Slightly warm	Spleen Lung	Invigorating spleen and strengthening middle energizer. Invigorating splenic yang.	Antifatigue Antistress Antiageing
	*Licorice*	Sweet Warm	Heart Lung Spleen Stomach	Invigorate spleen and nourishing qi. Relieving spasm and pain.	Antiarrhythmia Analgesia Adrenal cortical hormone-like effect

Blood tonics	*Radix Paeoniaalba*	Bitter Sour Slightly cold	Liver Spleen	Nourishing blood and retaining yin. Nourishing liver and relieving pain.	Anti-inflammation Analgesia
	*Angelica sinensis*	Sweet Pungent Warm	Liver Heart Spleen	Replenishing blood and regulating menstruation. Promoting blood circulation to arrest pain.	Dilate coronary artery Protect the myocardium
	*Polygonum multiflorum thumb*	Bitter Sweet Astringent Slightly warm	Liver Kidney	Strengthening and nourishing marrow and essence.	Antiageing
	*Radix Rehmanniae*	Sweet Slightly warm	Liver Kidney	Replenishing blood nourishing yin. Replenishing essence and nourishing marrow.	Promotes the synthesis of adrenal cortical hormone

Ying-invigorating	*Lily*	Sweet Slightly cold	Lung Heart Stomach	Nourishing yin and moistening lung. Cooling pericardium for tranquilization.	Composure Antiallergy
	*Glossy Privet Fruit*	Sweet Bitter Cool	Liver Kidney	Nourishing liver and kidney	Antiageing Regulates the balance of immunity
	*Eclipta alba*	Sweet Sour Cold	Liver Kidney	Nourishing liver and kidney. Cooling blood for hemostasis.	Improves immune function

Yang-invigorating	*Psoralea corylifolia*	Bitter Pungent Warm	Kidney Spleen	Invigorating kidney and strengthening yang. Consolidating essence and astringing urine.	Antiageing Neuromodulation
	*Morinda officinalis*	Pungent Sweet Slightly warm	Kidney Liver	Invigorating kidney and strengthening yang.	Strengthen adrenal cortical Hormone-like effect
	*Cistanche tubulosa*	Sweet Salty Warm	Kidney Large intestine	Invigorating kidney and strengthening yang.	Active adrenal gland to release corticosteroids
	*Folium Epimedii*	Sweet Pungent Warm	Liver Kidney	Invigorating kidney and strengthening yang. Dispelling wind to eliminate dampness.	Strengthens the endocrine function of thalamic-pituitary and adrenal cortex axis
	*Fructus Alpinia oxyphylla*	Pungent Warm	Kidney Spleen	Astringing urine. Warming spleen and promoting appetite.	Strengthen myocardial constriction

**Table 2:** Experimental research of neurotransmitters affected by tonic traditional Chinese medicine.

Condition	Products (classification)/formula	Effective components	Animals (weight/age)	Models^a^	Daily dose (duration)	Material parts	Neurotransmitters/relative receptors	Main^b^ results
Depression	*Rhodiola rosea* (Qi-invigorating)	Ethanol extract	Male SD rats 180–200 g	CMUS	1.5, 3, and 6 g/kg (3 weeks)	Hippocampus	5-HT level	5-HT↑

Depression	*Acanthopanax senticosus* (Qi-invigorating)	Aqueous extract	Male KM mice 18–20 g	FST TST	500, 1000, and 2000 mg/kg (7 days)	Brain	5-HT, NE, and DA	5-HT↑ NE↑ DA↑

Depression	*Ginseng* (Qi-invigorating)	20(S)-protopanaxadiol	Male Swiss mice 18–22 g Male SD rats 200–250 g	FST TST OB	3.75, 7.5, and 15 mg/kg (10 days) 13.33 and 6.67 mg/kg (14 days)	Hippocampus and cortex	NE and 5-HT	NE↑ and 5-HT↑

Depression	*Licorice* (Qi-invigorating)	Liquiritin and isoliquiritin	Mice	FST TST	10, 20, and 40 mg/kg	Hippocampus, hypothalamus, and cortex	5-HT and NE	5-HT↑ NE↑ 5-HIAA/5-HT↓

Depression	*Peony* (blood tonics)	Paeoniflorin H	Male SD rats 140–160 g	CMUS	30 and 60 mg/kg (4 weeks)	Brain	5-HT/5-HIAA, NA and DA	NA↑ DA↑ 5-HT↑ 5-HIAA/5-HT↓

Depression	*Psoralea corylifolia* (Yang-invigorating)	Psoralen	Male ICR mice 23–25 g,	FST	10, 20, and 40 mg/kg (14 days)	Frontal cortex and hippocampus	5-HT/5-HIAA	5-HT↑

AD	*Eclipta alba* (Ying-invigorating)	Aqueous extracts	Half male and female SD rats 220 ± 20 g	A*β*25–35- infused	8 and 24 g/kg (8 weeks)	Hippocampal	NE, DA, 5-HT, ChAT, and AChE	NE↑ DA↑ 5-Ht↑ChAT↑ AchE↓

AD	*Morinda officinalis* (Yang-invigorating)	Bajijiasu	Male SD rats 180–220 g,	A*β*25–35- infused	8, 24, and 48 mg/kg (25 days)	Brain	ACh, AchE NE, DA, 5-HT, and 5-HIAA	ACh↑ AchE↓ NE↑ DA↑ 5-HT↑

AD	*Cistanche Tubulosa* (Yang-invigorating)	Aqueous extracts	Male SD rats 300–350 g	A*β* 1–42-infused	100 and 200 mg/kg (15 days)	Cortical and hippocampal	ACh, DA AChE, and MAO-A	DA↑ AChE↓ MAO-A↓

PD	*Acanthopanax senticosus Harms* (Qi-invigorating)	Ethanol extract	Male C57BL/6 mice 18–22 g,	MPTP-treated	45.5 and 182 mg/kg (20 days)	Striatum	DA, HVA	DA↑

PD	*Panax quinqnefolism* (Qi-invigorating)	Pseudoginsenoside-F11	Male SD rats 200–250 g	6-OHDA-induced	3, 6, and 12 mg/kg (3 weeks)	Striatum	DA TH	DA↑ TH↑

PD	*Astragalus* (Qi-invigorating)	*Astragalus *polysaccharide	*C. elegans *strain BZ555.	6-OHDA-induced	1, 2, and 4 mg/mL	BZ555 nematodes	DA and AChE	DA↑ AChE↑

PD	*Peony* (blood tonics)	Paeoniflorin	Adult male C57BL/6 mice 25 g,;	MPTP-treated	7.5, 15, and 30 mg/kg (7 days)	Striatum	DA, HVA, DAT, and TH	DA↑ HVA↑ DAT↑ TH↑

PD	*Fallopia multiflora* (blood tonics)	2,3,5,4-Tetrahydroxystilbene-2-O-*β*-D-glucoside	Male C57BL/6 mice 22–25 g	MPTP-induced	20 and 40 mg/kg (14 days)	Striatum	DA, DOPAC, HVA, and TH	DA↑ DOPAC↑ HVA↑ TH↑

PD	*Fructus Alpinia oxyphylla* (Yang-invigorating)	Ethanolic extract	Zebrafish	6-OHDA-induced	6 and 12 *μ*g/ml (2 days)	Brain	DA	DA↑

PD	*Psoralea corylifolia* (Yang-invigorating)	△3,2-hydroxy bakuchiol	Male SD rats 200–220 g Male C57BL/6J mice 30–36 g	MPTP-induced	5.44, 16.32, and 48.96 mg/kg 4, 20, and 100 mg/kg	Striatum Hippocampal	DA, NE, and TH	DA↑ NE↑ TH↑

PD	Jia-Jian-Di-Huang-Yin-Zi decoction	*Radix Paeonia alba, Angelica sinensis, Radix Rehmanniae, Morinda officinalis, and Cistanche*	Male C57BL/6 mice 25–30 g	MPTP-induced	8.5, 17, and 34 g/kg (14 days)	Striatum	DOPAC, DA, and HVA	DA↑ DOPA↑ HVA↑

Sleep disorders	*Licorice *(Qi-invigorating)	Ethanol extract; glabrol	Male ICR mice 18–22 g Male C57BL/6 N mice 27–30 g	Pentobarbital-induced sleep	50, 100, 250, and 500 mg/kg 50 mg/kg	Cortex	GABAA-BZD receptors	GABAA-BZD↑

Sleep disorders	*Eleutherococcus senticosus* (Qi-invigorating)	Eleutheroside E	Male ICR mice 18–22 g	Sleep deprivation	50 mg/kg (10 days)	Hippocampus	5-HT and DA	5-HT↑ DA↓

Sleep disorders	*Alpinia oxyphylla fructus* (Yang-invigorating)	Volatile oil	Half male and female Wistar rats (200 ± 20) g	Sleep deprivation	9 and 27 g/kg (14 days)	Hypothalamus Cortex	Glu and GABA	Glu↓ GABA↑

Perimenopausal syndrome	A derivative herbal recipe from Danggui Buxue Tang	*Radix Astragali,Radix Ange, Llicae sinensis,* *Folium Epimedii*	Female SD rats (180 ± 20) g	OVX	141, 282, and 564 mg/kg (16 weeks)	Hypothalamuses	NE, DA, 5-HT, and 5-HIAA	NE↓ DA↓ 5-HT↓

Perimenopausal syndrome	Erzhibaihe Cream	*Glossy Privet Fruit, Eclipta alba,Lily*	Female Wistar rats (200 ± 20) g	OVX	0.48, 0.96 and 1.92 mg/kg (45 days)	Hypothalamus	NE, DA, and 5-HT	NE↓ DA↓ 5-HT↓

Perimenopausal syndrome	Bushen Liaogeng Extract (BLE)	*RadixRehmannia,* *Praeparata* *Cistanche* *Cynomorium songaricum*	Female SD rats 180–220 g	OVX	4.5, 9 and 18 g/kg (4 weeks)	Hypothalamus	DA, NE, 5-Ht, and 5-HIAA	DA↓ NE↓ 5-HT↓ 5-HIAA↓

*Note*. ^a^Models: CMUS: chronic mild unpredictable stress; FST: forced swimming test; TST: tail suspension test; OFT: open-field test; OB: olfactory bulbectomy; 6-OHDA: 6-hydroxydopamine; OVX: ovariectomy; ^b^main results↑: increased compared with model or control group; ^b^main results↓: decreased compared with model or control group.

## References

[1] Liu S.-S., Ai Q.-D., Chen N.-H. (2016). Reaearch Progress of Anti-Aging Active Ingredients of Tonic Chinese Medicine.

[2] Lv A.-P., Li D.-X., Lin Z.-R. (2005). The effects of supplemental spleen prescription on the activity of three antioxidant enzymes in different tissues of spleen deficiency rats.

[3] Qiu Y., Du G.-H., Qu Z.-W. (1996). Comparison of the anti-lipid peroxidative effects of some traditional Chinese nourishing-tonifying drugs in vitro.

[4] Xiao K., Miao M.-S. (2014). Related Research of Qi-tonifying Drugs.

[5] Hu F. (2017). Application and Analysis of Huang Yuanyu's Experience of Using Blood Tonic Medicine.

[6] Guan H.-Q., Tan F., Fan Q.-L. (2016). Exploration on Compatibility of Herbs for Nourishing Yin in Formulas of Kidney -yang Tonification.

[7] Tang J.-F. (2004). The discussion on the application of the syndrome differentiation of yang-tonifying drugs.

[8] Wang Z., Jian C., Chuan C. (2014). Effect of supplementing medicine and its effective components on learning and memory abilities in patients with Alzheimers disease.

[9] Song X.-Y., Chen L.-Y., Yan S.-Y. (2017). Discussion on relationship between yin-yang, neutralization and traditional Chinese medicine.

[10] Zhang S.-S. (2014). Thinking and cognition of the theory of yin and Yang in traditional Chinese Medicine.

[11] Liu Y.-J., He L., Long C. (2017). Chengxing Long, Discussion on Consistency of TCM Yin-Yang Balance and Microecological Balance.

[12] Xiao H.-Y., Ma Y.-D., Liu L.-L. (2012). Discussion on the origin of Qi in Chinese medicine in the perspective of philology.

[13] Yang K. (2011). Discussion on relationship between gas and blood and its clinical significance.

[14] Li H.-M., Wang X. (2016). Discussion on the theory of mutual assistance, balance, guard and harmony in qi and blood.

[15] Chen J.-D. (2010). Mechanism and medical model formation of meridians in circulating qi and blood.

[16] Chang H., Xie Y., Wen Y., Zhang S., Qu J., Lu W. (1983). Further Investigation on the Hypothesis of Meridian-Cortex-Viscera Interrelationship.

[17] Omura Y. (1989). Connections found between each meridian (heart, stomach, triple burner, etc.) & organ representation area of corresponding internal organs in each side of the cerebral cortex.

[18] Liu L.-Y., Pan J., Zhang H. (2002). The morphological structure of the skin along the meridian and the transmitting mechanism of arrector Pili muscle-sympathetic axon reflex.

[19] Liu L.-Y., Fan J.-Y. (1997). Study on the release of catecholamine along channels increased by acupuncture.

[20] Nutt D. J. (2008). Relationship of neurotransmitters to the symptoms of major depressive disorder.

[21] Xu C.-Y., Tian J.-Z., Shiet J. (2013). Study of the TCM Syndromes Characteristics of Depression.

[22] Bondi C. O., Rodriguez G., Gould G. G., Frazer A., Morilak D. A. (2008). Chronic unpredictable stress induces a cognitive deficit and anxiety-like behavior in rats that is prevented by chronic antidepressant drug treatment.

[23] Porsolt R. D., Le Pichon M., Jalfre M. (1977). Depression: a new animal model sensitive to antidepressant treatments.

[24] Steru L., Chermat R., Thierry B., Simon P. (1985). The tail suspension test: a new method for screening antidepressants in mice.

[25] Veenema A. H., Cremers T. I. F. H., Jongsma M. E., Steenbergen P. J., De Boer S. F., Koolhaas J. M. (2005). Differences in the effects of 5-HT1A receptor agonists on forced swimming behavior and brain 5-HT metabolism between low and high aggressive mice.

[26] Sha D., Jin H., Kopke R. D., Wu J. (2004). Choline Acetyltransferase: Regulation and Coupling with Protein Kinase and Vesicular Acetylcholine Transporter on Synaptic Vesicles.

[27] Szymański P., Janik A., Zurek E., Mikiciuk-Olasik E. (2011). Design, synthesis and biological evaluation of new 2-benzoxazolinone derivatives as potential cholinesterase inhibitors for therapy of Alzheimer's disease.

[28] Miner L. H., Jedema H. P., Moore F. W., Blakely R. D., Grace A. A., Sesack S. R. (2006). Chronic stress increases the plasmalemmal distribution of the norepinephrine transporter and the coexpression of tyrosine hydroxylase in norepinephrine axons in the prefrontal cortex.

[29] Terry A. V., Buccafusco J. J. (2003). The cholinergic hypothesis of age and Alzheimer's disease-related cognitive deficits: recent challenges and their implications for novel drug development.

[30] Xiao M.-M. (2012). Research Progress of Traditional Chinese Medicine for Alzheimer's disease.

[31] Parihar M. S., Hemnani T. (2004). Alzheimer's disease pathogenesis and therapeutic interventions.

[32] Tran M. H., Yamada K., Nabeshima T. (2002). Amyloid *β*-peptide induces cholinergic dysfunction and cognitive deficits: A minireview.

[33] Iversen L. L., Mortishire-Smith R. J., Pollack S. J., Shearman M. S. (1995). The toxicity in vitro of *β*-amyloid protein.

[34] Kaushik P., Gorin F., Vali S. (2007). Dynamics of tyrosine hydroxylase mediated regulation of dopamine synthesis.

[35] Zimmer L., Delion-Vancassel S., Durand G. (2000). Modification of dopamine neurotransmission in the nucleus accumbens of rats deficient in n-3 polyunsaturated fatty acids.

[36] Johnston G. A. R. (2005). GABAA receptor channel pharmacology.

[37] Bateson A. N. (2006). Further potential of the GABA receptor in the treatment of insomnia.

[38] Liu D.-E. (2004). Physiological and pathological changes of perimenopausal women.

[39] Li Z., Xue J., Chen P., Chen L., Yan S., Liu L. (2014). Prevalence of nonalcoholic fatty liver disease in mainland of China: a meta-analysis of published studies.

[40] Xie Q.-F., Xie J.-H., Dong T. T. X. (2012). Effect of a derived herbal recipe from an ancient Chinese formula, Danggui Buxue Tang, on ovariectomized rats.

[41] Yang S.-W., Guo J.-S., Wanget X.-Q. (2016). Kidney therapy more extracton castration menopause model neural endocrine dysfunction in rats.

[42] Fu G.-X., Zhao X.-H., Pan H.-T. (2009). The study of climacteric syndrome.

[43] Chen Q. G., Zeng Y. S., Qu Z. Q. (2009). The effects of *Rhodiola rosea* extract on 5-HT level, cell proliferation and quantity of neurons at cerebral hippocampus of depressive rats.

[44] Jin L., Wu F., Li X. (2013). Anti-depressant effects of aqueous extract from *Acanthopanax senticosus* in mice.

[45] Dhingra D., Sharma A. (2006). Antidepressant-like activity of *Glycyrrhiza glabra* L. in mouse models of immobility tests.

[46] Zhao Z.-Y., Wang W.-X., Guo H.-Z. (2006). Anti-depressive effect of liquiritin on chronic stress depression in rats.

[47] Wang W., Hu X., Zhao Z. (2008). Antidepressant-like effects of liquiritin and isoliquiritin from Glycyrrhiza uralensis in the forced swimming test and tail suspension test in mice.

[48] Dang H., Chen Y., Liu X. (2009). Antidepressant effects of ginseng total saponins in the forced swimming test and chronic mild stress models of depression.

[49] Xu C., Teng J., Chen W. (2010). 20(S)-protopanaxadiol, an active ginseng metabolite, exhibits strong antidepressant-like effects in animal tests.

[50] Hung H.-C., Lee E. H. Y. (1996). The mesolimbic dopaminergic pathway is more resistant than the nigrostriatal dopaminergic pathway to MPTP and MPP+ toxicity: Role of BDNF gene expression.

[51] Di Monte D. A. (2001). The role of environmental agents in Parkinson's disease.

[52] Liu S., Li X., Huo Y., Lu F. (2012). Protective effect of extract of *Acanthopanax senticosus* harms on dopaminergic neurons in Parkinson's disease mice.

[53] Tian X., Ren Y.-Y., Li P.-Y. (2012). Separation and Purification Technology of Pseudo-ginsenoside F11 from Total Saponins in Leaves and Stems of Panax quinquefolium.

[54] Wang J. Y., Yang J. Y., Wang F. (2013). Neuroprotective effect of pseudoginsenoside-F11 on a rat model of Parkinson's disease induced by 6-hydroxydopamine.

[55] Li W.-X., Sun S.-G., Yuan H., Wang H.-T., Zhang Y.-B., Sun B.-L. (2006). Time dependency of *astragalus* polysaccharides against systemic injury of free radical in astrocyte culture medium.

[56] Li H., Shi R., Ding F. (2016). Astragalus Polysaccharide Suppresses 6-Hydroxydopamine-Induced Neurotoxicity in Caenorhabditis elegans.

[57] Cho S., Park J.-H., Pae A. N. (2012). Hypnotic effects and GABAergic mechanism of licorice (Glycyrrhiza glabra) ethanol extract and its major flavonoid constituent glabrol.

[58] Doe J. (1999).

[59] Chinese Pharmacopoeia Section one.

[60] Huang L.-Z., Wei L., Zhao H.-F., Huang B.-K., Rahman K., Qin L.-P. (2011). The effect of Eleutheroside E on behavioral alterations in murine sleep deprivation stress model.

[61] Mao Q., Huang Z., Ip S., Che C. (2008). Antidepressant-like effect of ethanol extract from *Paeonia lactiflora* in mice.

[62] Mao Q.-Q., Ip S.-P., Ko K.-M., Tsai S.-H., Xian Y.-F., Che C.-T. (2009). Effects of peony glycosides on mice exposed to chronic unpredictable stress: further evidence for antidepressant-like activity.

[63] Qiu F.-M., Zhong X.-M., Mao Q.-Q., Huang Z. (2013). Antidepressant-like effects of paeoniflorin on the behavioural, biochemical, and neurochemical patterns of rats exposed to chronic unpredictable stress.

[64] Zheng M., Liu C., Fan Y., Yan P., Shi D., Zhang Y. (2017). Neuroprotection by Paeoniflorin in the MPTP mouse model of Parkinson's disease.

[65] He H., Wang S., Tian J. (2015). Protective effects of 2,3,5,4′-tetrahydroxystilbene-2-O-*β*-d-glucoside in the MPTP-induced mouse model of Parkinson's disease: Involvement of reactive oxygen species-mediated JNK, P38 and mitochondrial pathways.

[66] Wang A.-M., Geng R.-J., Li Y. (2016). Effect of Eclipta on Learning and Memory and Hippocampal Neurotransmitters in the Senile Dementia Model Rats.

[67] Chen Y., Kong L.-D., Xia X., Kung H.-F., Zhang L. (2005). Behavioral and biochemical studies of total furocoumarins from seeds of *Psoralea corylifolia* in the forced swimming test in mice.

[68] Xu Q., Pan Y., Yi L.-T. (2008). Antidepressant-Like Effects of Psoralen Isolated from the Seeds of Psoralea corylifolia in the Mouse Forced Swimming Test.

[69] Chen D.-L., Zhang P., Lin L. (2013). Effect of oligosaccharides from Morinda Officinal is on ß-amyloid-induced learning and memory dysfunction in rats.

[70] Liu X.-H., Xiao F.-X., Chen Y.-G. (2009). Determination of Bajijiasu in Morinda officinalis How by HPLC-ELSD.

[71] Chen D.-L., Zhang P., Lin L. (2014). Protective effects of bajijiasu in a rat model of A*β*25-35- induced neurotoxicity.

[72] Wu C.-R., Lin H.-C., Su M.-H. (2014). Reversal by aqueous extracts of *Cistanche tubulosa* from behavioral deficits in Alzheimer's disease-like rat model: relevance for amyloid deposition and central neurotransmitter function.

[73] Zhang Z.-J., Cheang L. C. V., Wang M.-W. (2012). Ethanolic extract of fructus *Alpinia oxyphylla* protects against 6-hydroxydopamine-induced damage of PC12 cells in vitro and dopaminergic neurons in zebrafish.

[74] Chen Y., Wu Y., Song J.-C. (2016). Research progress of the chemical constituents and bioactivity of Psoralea corylifolia.

[75] Zhao G., Zheng X.-W., Qin G.-W., Gai Y., Jiang Z.-H., Guo L.-H. (2009). In vitro dopaminergic neuroprotective and in vivo antiparkinsonian-like effects of Δ^3^,2-hydroxybakuchiol isolated from *Psoralea corylifolia* (L.).

[76] Cui K.-Y., Wang P., You Q.-Y. (2013). Effects of Volatile Oil Extracted from Alpinia oxyphylla Fructus on Amino Acid Neurotransmitters and its Receptor Expressionin Brain of the Model Rats with Sleep Deprivation Recovery.

[77] Zhang J., Zhang Z., Bao J. (2017). Jia-Jian-Di-Huang-Yin-Zi decoction reduces apoptosis induced by both mitochondrial and endoplasmic reticulum caspase12 pathways in the mouse model of Parkinson's disease.

